# Evaluation of HIF-1α and VEGF-A expression in radiation-induced cystitis: A case-control study

**DOI:** 10.1590/S1677-5538.IBJU.2020.0054

**Published:** 2021-02-03

**Authors:** Ricardo Castellani de Mattos, Isabella dos Santos Guimarães, Leandro de Souza Thiago, Andreia Cristina de Melo

**Affiliations:** 1 Instituto Nacional do Câncer Divisão de Pesquisa Clínica Rio de Janeiro RJ Brasil Divisão de Pesquisa Clínica, Instituto Nacional do Câncer - INCA, Rio de Janeiro, RJ, Brasil.

**Keywords:** Neoplasms, Radiation-Induced, Uterine Cervical Neoplasms, Vascular Endothelial Growth Factor A

## Abstract

The standard treatment for locally advanced cervical cancer (CC) is chemoradiotherapy. Once the bladder receives part of the radiation, a typical inflammatory condition that configures radiation-induced cystitis may develop. Chronic radiation-induced cystitis is commonly characterized by the bladder new submucosal vascularization, which is typically fragile and favors hematuria. The current study aims to investigate if Hypoxia-Induced Factor (HIF-1α) and its transcriptional target Vascular Endothelial Growth Factor A (VEGF-A) could be a primary pathway leading to increased submucosal vascularization. HIF-1α and VEGF-A mRNA levels in bladder core biopsies from CC patients treated with radiotherapy versus untreated (non-irradiated) patients were analyzed using a droplet digital polymerase chain reaction technology. Gene expression results showed that HIF-1α and VEGF-A had no significant differences between bladder samples from patients previously irradiated and untreated patient samples. However, a direct relationship between the degree of late morbidity and the expression of HIF-1α and VEGF-A has been demonstrated. Despite the lack of statistical significance precludes a definitive conclusion, the data presented herein suggests that further studies investigating the role of HIF-1α in bladder neovascularization in radiation-induced cystitis are highly recommended.

## INTRODUCTION

Cervical cancer (CC) is the fourth most common malignancy diagnosed in women worldwide. In Brazil, CC is the third most common cancer and the third cause of cancer death in women (1). The standard treatment for locally advanced CC is chemo-radiotherapy. Once the bladder receives part of the radiation, a typical inflammatory condition that configures radiation––induced cystitis (actinic cystitis) may develop.

The reported incidence of late actinic cystitis that are related to pelvic neoplasms radiotherapy varies from 5 to 10% (2). However, radiation-induced cystitis has already been estimated to range between 8 and 12% in CC patients, with moderate or severe sequelae ranging between 2 and 6% (3). Levenback et al. reported an incidence of 6.5% in 1.784 stage IB CC patients treated with radiotherapy over a period of 29 years (4).

Radiation-induced cystitis can be chronologically divided into three distinct phases: (i) an acute phase that begins during treatment and improves within several weeks after the course of radiotherapy is completed, (ii) an asymptomatic latent phase, dose––dependent, which may last months or even years, and (iii) a chronic phase of late and irreversible response. Chronic irradiated tissues are commonly characterized by hypoxia. The capacity of tissue repair is severely compromised and may result in fibrosis, ulceration and fistula. The bladder new submucosal vascularization, which is typically fragile, favors hematuria (5).

Therefore, Hypoxia-Induced Factor (HIF-1α) and its transcriptional targets, including Vascular Endothelial Growth Factor A (VEGF-A) could be a primary pathway which leads to increased submucosal vascularization in the radiation––damaged bladder. HIF-1α is a transcription factor that, after a hypoxia insult, associates with specific nuclear cofactors, activating the specific genes that are responsible for various adaptive responses to low oxygen tensions (6).

Radiation cystitis is a challenging problem for urologists and the effectiveness of therapies is limited due in part to the lack of understanding of the molecular changes that are responsible for the progression of the disease. The current study aims to compare the expression of HIF-1α and VEGF-A in bladder tissue samples from patients undergoing radiotherapy as a treatment for CC versus untreated (non-irradiated) patients.

## MATERIAL AND METHODS

### Study design

A single-institution, case-control study was performed in CC patients that were previously treated with pelvic radiotherapy versus non-irradiated CC patients. Tissue fragments were collected by endoscopic bladder biopsy. Control patients were led to participate in the study during the staging exam and irradiated patients were then invited after consulting the medical records or before carrying out a previously scheduled exam, mostly due to chronic hematuria.

This study was approved by the Ethics in Human Research Committee of the Brazilian National Cancer Institute (INCA), Rio de Janeiro, Brazil (number 918.354), and was conducted in accordance with the Good Clinical Practice Guidelines. All patients gave written informed consent.

### Eligibility

Patients older than 18 years of age with primary squamous cell carcinoma or adenocarcinoma of cervix, performance status (PS) of 0, 1, or 2 and proper organic function were eligible to be included in the study. All medical records were available for data collection purposes. Patients with blood clotting disorder, active macroscopic hematuria or active uri-nary tract infection as clinically demonstrated or in laboratory tests were excluded.

Patients selected for irradiated group should have been treated for CC with external radiotherapy from August 2010 to June 2016, respecting an interval between the completion of external radiotherapy and cystoscopy of no less than 18 months. The time chosen between radiotherapy and sample collection had to correspond to the installed chronic actinic damage. If the interval was too short it would be feasible that the findings would correspond to acute injury. Since chronic damage is irreversible, there was no maximum interval between treatment and sample collection.

Patients that were both diagnosed with CC and treatment-free and whose clinical staging was no more advanced than IIIA were included in the control group.

### Patient data collection

All relevant demographic, clinical and laboratory data, histological type, staging and radiation doses were collected from the medical charts and a bladder endoscopic examination was performed.

Regarding late adverse events, patients were evaluated according to the Common Terminology Criteria for Adverse Events (CTCAE) system, version 4.03 (7). During endoscopic evaluation the presence of actinic cystitis was classified according to the Radiation Therapy Oncology Group/European Organization for Research and Treatment of Cancer (RTOG/EORTC) system. This considers symptoms and endoscopic findings (8) and the evaluation was also performed according to the Late Effects Normal Tissue Task Force/Subjective, Objective, Management and Analytic (LENT/SOMA) system (9).

Sampling, handling and storage of bladder core biopsy and bladder washing for molecular assays.

A (very) small fragment of bladder tissue was removed by cystoscopy. In general terms, the preferred puncture site in non-irradiated women was the posterior vesical wall while in previously irradiated women, for safety reasons, the elected puncture site was the less vascularized one.

In some individuals from the control group, bladder washing samples were collected by barbotage before core biopsy, kept at 4°C and then sent to the laboratory to be promptly processed. Tissue samples were immediately placed on RNAlater™ Tissue Collection (Thermo Fisher Scientific, Wilmington, DE, USA), left at 4°C for 24 hours, then, the RNAlater™ was discarded and samples were stored dry at −80°C until RNA isolation.

### RNA extraction and complementary DNA synthesis

Tissue homogenization and subsequent RNA isolation were performed using the Omni TH tissue homogenizer® (Omni International, Kennesaw, GA, USA) in combination with the RNeasy Mini Kit® (Qiagen, Hilden, Germany). The RNA isolation was performed according to the protocol of the manufacturer. All RNA samples were stored at −80°C prior to further use. RNA concentrations were determined by OD 260nm measurements on a NanoDrop ND-1000 spectrophotometer® (Thermo Fisher Scientific, Wilmington, DE, USA). RNA was reverse transcribed using Superscript II reverse transcriptase® (Invitrogen, Carlsbad CA, USA) according to the manufacturer's protocol.

### Droplet digital PCR assays for VEGF-A and HIF-1α

Droplet digital PCR (ddPCR) using TaqMan™ hydrolysis probes chemistry was performed using a Bio-Rad QX100 Droplet Digital PCR system (Bio-Rad, Pleasanton, CA, USA). Bio-Rad QX100 reagents and consumables were used for the experiments. Quantitative polymerase chain reaction assays were performed using HIF1α-FAM and VEGF-A-FAM TaqMan assays. Glyceraldehyde-3-phosphate dehydrogenase (GAPDH)-HEX assays were performed for each sample on each plate for normalization of all other targets. Reactions were performed in appropriate volumes using ddPCR 2x Master Mix, 20x TaqMan Probe Mix, nuclease free water and reverse transcriptase product. Each ddPCR assay mixture was dispersed into droplets using the QX100 droplet generator (Bio-Rad, Pleasanton, CA, USA). When droplet generation was completed, the droplets were transferred to a 96-well PCR plate and heat-sealed with sealing foil sheets (Bio-Rad, Pleasanton, CA, USA). The PCR amplification was performed in a sealed 96-well plate using a Veriti™ thermal cycler (Thermo Fisher Scientific, Wilmington, DE, USA) with the following cycling parameters: 95°C for 10 minutes followed by 40 cycles of 94°C for 30 seconds, 60°C for 70 seconds (ramp rate set to 2°C/s) and a final extension step at 98°C for 10 minutes and a hold at 4°C. After PCR is complete, the 96-well plate was loaded into the QX100 Droplet Reader and the QuantaSoft™ software version 1.5.38.118 (Bio-Rad, Pleasanton, CA, USA) was used for data analysis following manufacturer's recommendations. Each well was measured for fluorescence and the results were displayed as dot plots.

### Statistical Analyses

The Mann-Whitney U test was used to estimate the statistical significance of differences observed between case and control groups (Graph-Pad Prism 5.0 software), p values ≤0.05 were considered to be associated with statistical significance.

## RESULTS

Between February 2015 and July 2018, 14 previously irradiated patients and 12 patients as a control group were included. Clinical data from all patients are summarized in Table-1.

**Table 1:** Clinical characteristics of patients.

		Irradiated - n (%)	Control - n (%)
Age (years, median)		48, 31 - 66	44.5, 33 - 66
	brown	6 (42.9)	5 (41.7)
Color/race	white	7 (50.0)	4 (33.3)
	black	1 (7.1)	3 (25.0)
Histopathology	squamous cell carcinoma	12 (85.7)	11 (91.7)
	adenocarcinoma	2 (14.3)	1 (8.3)
	I	0	1 (8.3)
Grade	II	13 (92.9)	7 (58.3)
	III	1 (7.1)	3 (25)
	Not informed	0	1 (8.3)
	IB1	2 (14.3)	1 (8.3)
	IB2	1 (7.1)	1 (8.3)
FIGO stage at diagnosis*	IIA	1 (7.1)	1 (8.3)
	IIB	9 (64.3)	8 (66.7)
	IIIA	0	1 (8.3)
	IIIB	1 (7.1)	0
Total		14	12

* FIGO: International Federation of Gynecology and Obstetrics

The primary complication that was expected from the sample collection was hematuria. However, it should be emphasized that there were no complications related to the sample collection in any patient from either group.

The characteristics related to the treatment of irradiated patients and possible complications of radiotherapy and bladder toxicity scores are shown in Table-2. The external radiotherapy dose employed in 12 cases was 4500cGy, while two patients received 5040cGy. The external radiotherapy dose that reached the bladder, calculated when the three-dimensional conformational technique was used, did not reach the maximum restrictive value of 6500cGy for all cases (10). Eleven patients received 2400cGy dose during brachytherapy and two received 2800cGy. The total dose of radiotherapy employed, summing the dosages of external radiotherapy and brachytherapy, ranged from 6900 to 7840cGy, with an average of 7044cGy. The interval between the completion of external radiotherapy and biopsy cystoscopy ranged from 19 to 69 months, with an average of 46 months.

**Table 2:** Characteristics of the radiotherapy regimen employed, bladder toxicity score and radiotherapy-related complications.

Patient ID	External Radiotherapy				Bladder	Dosec	Brachytherapy		Total Dosec	Proctopathye	RTOG/EORTC			LENT/SOMA				CTCAE	Sediment^f^
	Equipmenta	Techiniqueb	Dosec	td	Average	Maximum	Insertion	Dosec				S	O	M	A	Maximum	Total		
101	LA	2D	4500	51	-	-	3	2400	6900	N	1	2	1	0	0	2	3	4	mild hemoglobinuria. moderate leukocyturia
102	cobalt	3D	4500	49	4806	4960	-	-	-	Grade 1	2	2		0	0	2	3	5	N
103	LA	2D	4500	49	-	-	3	2400	6900	N	1	0	1	0	0	1	1	0	N
104	LA	2D	4500	49	-	-	3	2400	6900	S	1	3	1	0	0	3	4	2	mild leukocyturia
105	LA	3D	4500	54	4730	4840	3	2400	6900	S	1	0	1	0	0	1	1	1	N
106	LA	3D	4500	54	4590	4680	4	2400	6900	N	1	0	1	0	0	1	1	2	moderate leukocyturia
107	LA	3D	4500	49	4700	4890	4	2800	7300	Grade 2	1	0	1	0	0	1	1	1	mild leukocyturia. mild proteinuria
108	LA	3D	4500	19	4384	4860	3	2400	6900	N	3	3	3	2	0	3	8	6	very intense hemoglobinuria, mild proteinuria
109	LA/cobalt	2D	5040	52	-	-	3	2400	7440	N	3	1	2	1	2	2	6	3	N
110	LA	3D	5040	49	5510	5580	4	2800	7840	N	1	2	1	1	0	2	4	4	mild proteinuria
111	LA	3D	4500	40	4610	4690	3	2400	6900	S	2	0	2	0	0	2	2	2	mild hemoglobinuria. mild proteinuria. intense leukocyturia
112	LA	3D	4500	69	4798	4850	4	2400	6900	S (diarrhea)	2	2	2	0	0	2	4	3	N
114	LA	2D	4500	24	-	-	3	2400	6900	N	2	2	2	1	0	2	5	3	intense hemoglobinuria. moderate proteinuria
115	cobalt	3D	4500	36	4820	4960	3	2400	6900	S	3	3	2	2	0	3	7	6	Not available
Mean			4557	46				2461	7044		1.64						3.57	3	

a- radiation source apparatus: LA - linear accelerator, cobalt - cobalt therapy;

b - technique used: 2D - two dimensions, 3D - three dimensions;

c-dose cGy;

d -t: interval between the end of external radiotherapy and cystoscopy with biopsy in months;

e - actinic proctopathy graduation based on the study site routine; N: normal examination, S: alteration present (grade not specified in rectosigmoidoscopy examination);

f urinary sediment; N: normal examination.

The RTOG/EORTC, LENT/SOMA and CTCAE scores, urinary sediment examination and presence of associated actinic rectal disease were considered. Fifty percent of irradiated patients had complaints compatible with rectal actinic damage, from which it was possible to endoscopically prove the complication in six patients and one had chronic diarrhea. On the RTOG/EORTC scale the score found seven patients with grade 1 toxicity, four patients with grade 2 and three patients with grade 3. The score for LENT/SOMA scale, considering the sum of toxicity grades, ranged from 1 to 8 (average 3.57). The sum of the degrees of severity on the CTCAE version 4.03 scale, including the different adverse events most likely related to bladder irradiation, ranged from 0 (one patient) to 6 (two patients), with a mean of 3.

**Figure 1:**
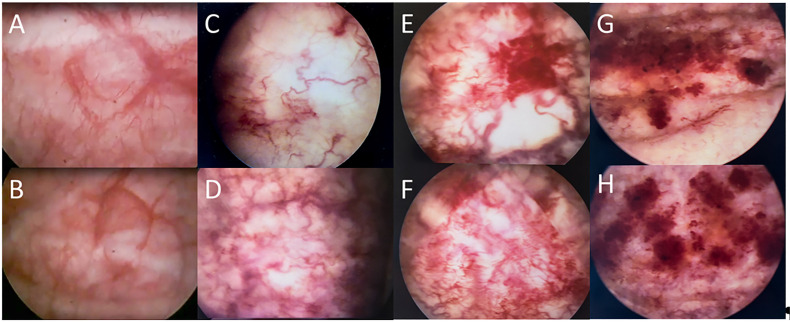
Aspect of the bladder mucosa of irradiated patients with varying degrees of neovascularization comparing with normal bladder mucosa. Images were obtained during cystoscopy examinations of patients included in the study and correspond to the endoscopic findings of RTOG/EORTC late morbidity scale: A and B - normal bladder mucosa, C and D -Grade 1: slight epithelial atrophy, minor telangiectasia, E and F - Grade 2: generalized telangiectasia, G and H – Grade 3: severe generalized telangiectasia (often with petechiae).

To further explore macroscopic changes of the urinary bladder associated with late radiation injuries, bladder endoscopic examination was performed. Bladder assessment by cystoscopy revealed varied intensities of radiation-induced damage according to the late RTOG/EORTC morbidity scale (Figure-1).

In light of the important role of hypoxia in increase submucosal vascularization in the radiation––damaged bladder we then evaluated the possible involvement of HIF-1α and its transcriptional target, VEGF-A, in bladder tissue samples from CC patients undergoing radiotherapy versus untreated (non-irradiated) CC patients.

**Figure 2:**
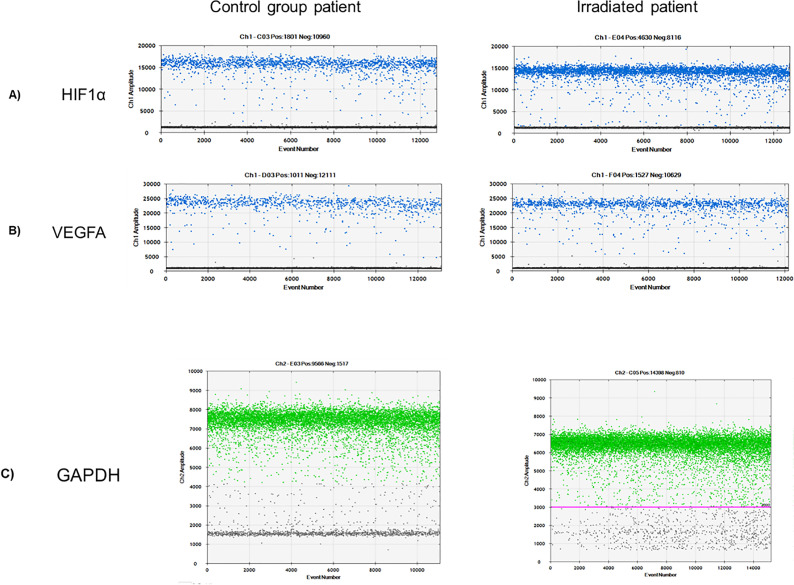
Graphics of digital PCR in drops. The graphs indicate the fluorescence intensity (Ch1 Amplitude and Ch2 Amplitude for FAM or HEX, respectively) of the drops analyzed (events number). The drops are termed "positive" or "negative" based on their fluorescence amplitudes. Positive events are marked in blue (HIF-1α and VEGF-A) and green (GAPDH), while negative events in gray (drops without the DNA molecule of interest). All analyzes were performed using QuantaSoft software (Biorad). During drop formation cDNA molecules are randomly distributed in the droplets. Due to the high number of independent events, the Poisson algorithm is used to determine absolute quantitation of copy number independent of a standard curve, allowing accurate quantification of gene expression. (A) Expression of HIF1α in a control group patient (left) and in an irradiated patient (right). (B) Expression of VEGFA in a control group patient (left) and in an irradiated patient (right). (C) Expression of GAPDH in a control group patient (left) and in an irradiated patient (right).

Total RNA extraction from irradiated group was performed in eight patients for molecular analysis and all patients of control group were included. Subsequently, gene expression analysis of HIF-1α, VEGF-A and GAPDH was performed by ddPCR using 5ng RNA for each reaction. As shown in Figure-2 it was possible to accurately quantify the gene expression of all targets in the irradiated patient samples and controls.

**Figure 3:**
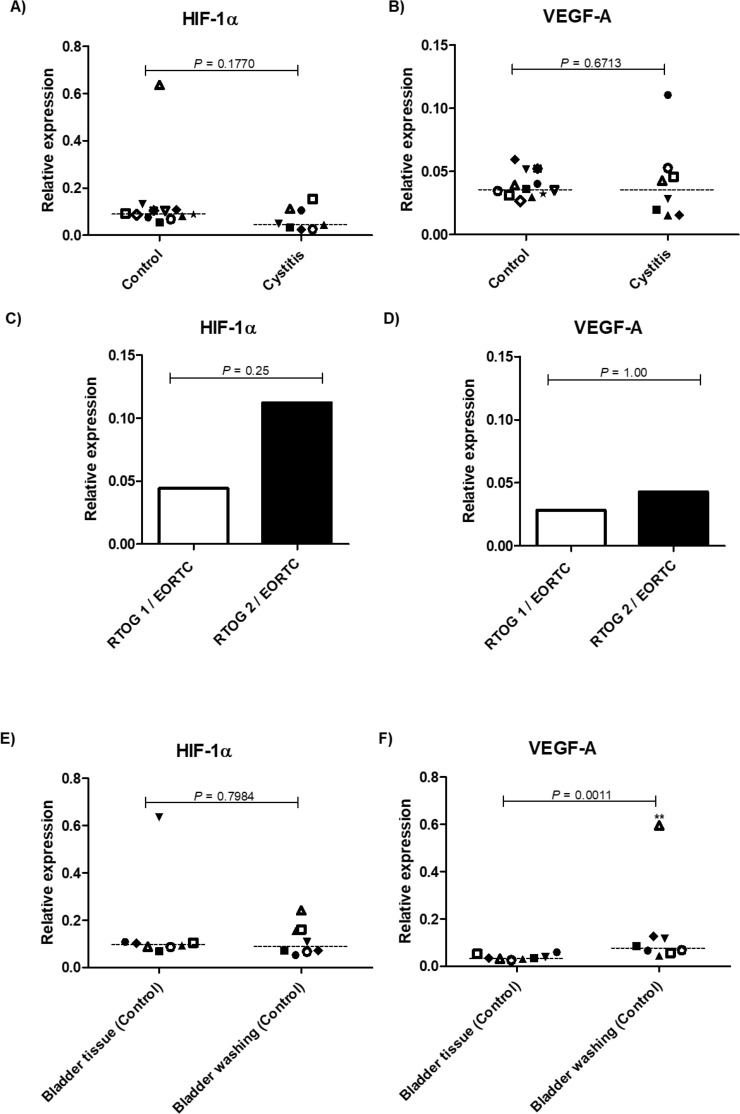
Droplet digital PCR expression analysis of HIF-1α and VEGF-A mRNA. (A) and (B) Analysis of HIF-1α and VEGF-A mRNA in non-irradiated patients (control) and patients previously submitted to pelvic radiotherapy (cystitis). (C) and (D) Analysis of HIF-1α and VEGF-A mRNA expression in patients previously submitted to pelvic radiotherapy according to the late RTOG/EORTC morbidity score. (E) and (F) Analysis of HIF-1α and VEGF-A mRNA in bladder tissue samples and washing bladder samples of non-irradiated patients. The targets mRNA expression were calculated relative to a stable expressed reference gene (GAPDH). Results were expressed as median and comparison between groups was performed by Mann–Whitney U test. RTOG/EORTC: grade 1 - mild epithelial atrophy, small telangiectasia (microscopic hematuria); grade 2 -moderate frequency, generalized telangiectasia, and intermittent macroscopic hematuria.

The gene expression results showed that HIF-1α had no significant differences between bladder samples from patients previously irradiated and untreated patient samples (p=0.177). Similar results were found in expression of VEGF-A (p=0.6713) (Figure-3).

Due to problems in the RNA extraction of patients with Grade 3 symptoms using the RTOG/EORTC scoring system, we only performed the analysis of HIF-1α and VEGF-A using the Grades 1 and 2. A direct relationship between the degree of late morbidity and the expression of HIF-1α and VEGF-A has been demonstrated (Figure-3). However, this trend did not reach statistical significance (p=0.250 and 1.0 respectively).

Considering the risks involving bladder biopsy in patients with radiotherapy-induced cystitis, in some individuals, from the control group, bladder washing samples were collected. As shown in Figure-3, there were similar expression levels of HIF-1α in bladder biopsy compared with washing samples of control group. However, the ddPCR showed that the mean relative expression of VEGF-A was significantly lower in bladder biopsy when compared with the washing samples of control group (p=0.0011).

## DISCUSSION

Hypoxia is a common phenomenon in the majority of cancers and great interest on HIF-1α stu-dies comes from its potential for targeted therapy through small inhibitors.

The current results on the expression pattern of HIF-1α and VEGF-A in bladder tissue from CC patients undergoing radiotherapy were in contrast to previous studies under other clinical or experimental conditions (11–18). Here, no differences on HIF-1α or VEGF-A mRNA levels were observed between irradiated versus non-irradiated groups (Figure-3). Moreover, we have also found that patients with higher RTOG/EORTC toxicity score exhibited a trend toward higher HIF-1α mRNA levels, in a directly proportional way.

Previous studies have shown an increased expression of HIF-1α, VEGF-A, and other angio-genesis markers in bladder cancer (11, 12). The relationship of HIF-1α and VEGF-A with radio-therapy-induced intestinal damage in prostate cancer or CC were also evaluated (13). The authors demonstrated in rectal biopsy samples a significant increase in HIF-1α (6.9-fold) and VEGF-A (4.7-fold) expression, as well as fibroblast-2 and interleukin-1β growth factor (13).

The expression of HIF-1α and VEGF-A and other markers of hypoxia, oxidative stress, angiogenesis and capillary proliferation, macrophage activation, cell signaling and fibrosis have also been studied in irradiated rat lungs (14). The authors verified the progressive increase of HIF-1α from 4 weeks and VEGF-A from 6 weeks after irradiation (14). Also, an increased expression of HIF-1α, HIF-2α and VEGF-A has been shown in bladder obstruction (15, 16), overactive bladder syndrome (17) and interstitial cystitis (18).

Next, an analysis was performed using the washing bladder samples of control group to test if those samples could provide a good model of radio-therapy-induced cystitis. Bladder washing exfoliates large sheets of urothelium and even three-dimensional urothelial fragments, however, we observed only a few preserved cells were recorded. The data showed that HIF-1α expression levels were similar in bladder biopsy compared with washing samples of control group. In contrast, VEGF-A mRNA levels were significantly lower in bladder biopsy when compared with washing samples of the control group. Clearly, additional analysis with more patients of the control and irradiated groups are needed to test if bladder washing samples could be a better approach for studying radiotherapy-induced cystitis.

It is important to point out, that the current study faced some limitations that could easily influence its findings. In addition to the difficulties in enrolling patients, an important issue was the quality and amplifiability of RNA in some samples. Samples collected in the early stages of the research may have experienced some level of RNA degradation. It should be noted that all samples collected were preserved in RNAlater® solution shortly after collection, in accordance with the manufacturer's recommendations. Other important hypotheses of note are the possibility of contamination due to the presence of blood cells in the biopsy material and the potential interference of tissue hypoxia on GAPDH expression (19). In light of this, a previous study has shown modulation of GAPDH mRNA expression after ionizing radiation (20).

Additionally, considering the risks involved in bladder biopsies in patients with radiotherapy-induced cystitis, obtaining samples in the most intensely vascularized patients was not considered safe by the investigators; therefore, the most affected areas were not represented in this trial. The obtained samples, in turn, also presented reduced dimensions, which made their processing and RNA extraction even more challenging.

It was also not possible to include patients irradiated in a single center or through a single technique. Thus, eventual differences in tissue hypoxia intensity and HIF-1α and VEGF-A expression between different external radiotherapy techniques may have resulted in differences in the detected values.

Most importantly, several of the limitations listed above are related to the fact that there is no other study that evaluated the expression of HIF-1α or VEGF-A in irradiated bladders.

## CONCLUSIONS

No differences on HIF-1α or VEGF-A mRNA levels were observed between the irradiated group versus the non-irradiated group. Among the irradiated patients, a direct proportional trend in the degree of RTOG/EORTC toxicity score and HIF-1α mRNA relative expression was observed, as HIF-1α mRNA levels increased alongside toxicity degree.

Although the lack of statistical significance precludes a definitive conclusion, the data presented herein suggests that further studies (with a higher number of patients) investigating the role of HIF-1α in bladder neovascularization in radiation-induced cystitis are highly recommended, given that radiation-induced cystitis is an orphan disease.

## ABBREVIATIONS

CC: cervical cancer
cGy: centigray
CTCAE: Common Terminology Criteria for Adverse Events
ddPCR: droplet digital polymerase chain reaction
GAPDH: Glyceraldehyde-3-phosphate dehydrogenase
HIF-1α: hypoxia-induced factor-1 α
INCA: Brazilian National Cancer Institute
mRNA: messenger ribonucleic acid
PS: performance status
RTOG/EORTC: The Radiation Therapy Oncology Group/ European Organisation for Research and Treatment of Cancer
VEGF-A: vascular endothelial growth factor A

## References

[1] 1. De Olivera Santos M. Estimativa 2018: incidência de câncer no Brasil - Instituto Nacional de Câncer José Alencar Gomes da Silva. Coordenação de Prevenção e Vigilância. [Internet]. Available at. <https://rbc.inca.gov.br/revista/index.php/revista/article/view/115>

[2] 2. Denton AS, Clarke NW, Maher EJ. Non-surgical interventions for late radiation cystitis in patients who have received radical radiotherapy to the pelvis. Cochrane Database Syst Rev. 2002;(3):CD001773. 10.1002/14651858.CD001773 PMC7025765 12137633

[3] 3. Marks LB, Carroll PR, Dugan TC, Anscher MS. The response of the urinary bladder, urethra, and ureter to radiation and chemotherapy. Int J Radiat Oncol Biol Phys. 1995;31:1257-80. 10.1016/0360-3016(94)00431-J 7713787

[4] 4. Levenback C, Eifel PJ, Burke TW, Morris M, Gershenson DM. Hemorrhagic cystitis following radiotherapy for stage Ib cancer of the cervix. Gynecol Oncol. 1994;55:206-10. 10.1006/gyno.1994.1278 7959285

[5] 5. Stewart FA. Mechanism of bladder damage and repair after treatment with radiation and cytostatic drugs. Br J Cancer Suppl. 1986;7:280-91. PMC2149791 3521706

[6] 6. Greer SN, Metcalf JL, Wang Y, Ohh M. The updated biology of hypoxia-inducible factor. EMBO J. 2012;31:2448-60. 10.1038/emboj.2012.125 PMC3365421 22562152

[7] 7. Common Terminology Criteria for Adverse Events (CTCAE) Version 4.0. (v4.03: 14, 2010) U.S. Department of Health and Human Services National Institutes of Health National Cancer Institute. [Internet]. Available at. https://ctep.cancer.gov/protocolDevelopment/electronic_applications/ctc.htm

[8] 8. Cox JD, Stetz J, Pajak TF: Toxicity criteria of the Radiation Therapy Oncology Group (RTOG) and the European organization for research and treatment of cancer (EORTC). Int J Radiat Oncol Biol Phys. 1995; 31 5: 1341–1346. 10.1016/0360-3016(95)00060-C 7713792

[9] 9. [No Authors]. LENT SOMA tables. Radiother Oncol. 1995;35:17-60. 7569012

[10] 10. Marks LB, Yorke ED, Jackson A, Ten Haken RK, Constine LS, Eisbruch A, et al. Use of normal tissue complication probability models in the clinic. Int J Radiat Oncol Biol Phys. 2010;76(3 Suppl):S10-9. 10.1016/j.ijrobp.2009.07.1754 PMC4041542 20171502

[11] 11. Ioachim E, Michael M, Salmas M, Michael MM, Stavropoulos NE, Malamou-Mitsi V. Hypoxia-inducible factors HIF-1alpha and HIF-2alpha expression in bladder cancer and their associations with other angiogenesis-related proteins. Urol Int. 2006;77:255-63. 10.1159/000094819 17033215

[12] 12. Tickoo SK, Milowsky MI, Dhar N, Dudas ME, Gallagher DJ, Al-Ahmadie H, et al. Hypoxia-inducible factor and mammalian target of rapamycin pathway markers in urothelial carcinoma of the bladder: possible therapeutic implications. BJU Int. 2011;107:844-9. 10.1111/j.1464-410X.2010.09517.x 20707797

[13] 13. Traub F, Schleicher S, Kirschniak A, Zieker D, Kupka S, Weinmann M, et al. Gene expression analysis in chronic postradiation proctopathy. Int J Colorectal Dis. 2012;27:879-84. 10.1007/s00384-011-1387-1 22173715

[14] 14. Rabbani ZN, Mi J, Zhang Y, Delong M, Jackson IL, Fleckenstein K, et al. Hypoxia inducible factor 1alpha signaling in fractionated radiation-induced lung injury: role of oxidative stress and tissue hypoxia. Radiat Res. 2010;173:165-74. 10.1667/RR1816.1 PMC2834297 20095848

[15] 15. Koritsiadis G, Tyritzis SI, Koutalellis G, Lazaris AC, Stravodimos K. The effect of alpha-blocker treatment on bladder hypoxia inducible factor-1 alpha regulation during lower urinary tract obstruction. Int Braz J Urol. 2010;36:86-94. 10.1590/s1677-55382010000100013 20202240

[16] 16. Iguchi N, Malykhina AP, Wilcox DT. Inhibition of HIF Reduces Bladder Hypertrophy and Improves Bladder Function in Murine Model of Partial Bladder Outlet Obstruction. J Urol. 2016;195(4 Pt 2):1250-6. 10.1016/j.juro.2015.08.001 PMC5505069 26926557

[17] 17. Christiaansen CE, Sun Y, Hsu YC, Chai TC. Alterations in expression of HIF-1α, HIF-2α, and VEGF by idiopathic overactive bladder urothelial cells during stretch suggest role for hypoxia. Urology. 2011;77:1266.e7-11. 10.1016/j.urology.2010.12.041 PMC3087847 21397301

[18] 18. Lee JD, Lee MH. Increased expression of hypoxia-inducible factor-1α and vascular endothelial growth factor associated with glomerulation formation in patients with interstitial cystitis. Urology. 2011;78:971.e11-5. 10.1016/j.urology.2011.05.050 21813166

[19] 19. Zhong H, Simons JW. Direct comparison of GAPDH, betaactin, cyclophilin, and 28S rRNA as internal standards for quantifying RNA levels under hypoxia. Biochem Biophys Res Commun. 1999;259:523-6. 10.1006/bbrc.1999.0815 10364451

[20] 20. Iyer G, Wang AR, Brennan SR, Bourgeois S, Armstrong E, Shah P, et al. Identification of stable housekeeping genes in response to ionizing radiation in cancer research. Sci Rep. 2017;7:43763. 10.1038/srep43763 PMC5338320 28262749

